# Functional alterations of myeloid cells during the course of Alzheimer’s disease

**DOI:** 10.1186/s13024-018-0293-1

**Published:** 2018-11-13

**Authors:** Aaron D. Thome, Alireza Faridar, David R. Beers, Jason R. Thonhoff, Weihua Zhao, Shixiang Wen, Belen Pascual, Joseph C. Masdeu, Stanley H. Appel

**Affiliations:** Department of Neurology, Houston Methodist Neurological Institute, 6560 Fannin St. Suite 802, Houston, TX 77030 USA

**Keywords:** Alzheimer’s disease, Inflammation, Myeloid cells, Monocyte, Microglia, Myeloid-derived suppressor cells

## Abstract

**Background:**

Neuroinflammation is a hallmark of neurodegenerative disease and a significant component of the pathology of Alzheimer’s disease (AD). Patients present with extensive microgliosis along with elevated pro-inflammatory signaling in the central nervous system and periphery. However, the role of peripheral myeloid cells in mediating and influencing AD pathogenesis remains unresolved.

**Methods:**

Peripheral myeloid cells were isolated from peripheral blood of patients with prodromal AD (*n* = 44), mild AD dementia (*n* = 25), moderate/severe AD dementia (*n* = 28), and age-matched controls (*n* = 54). Patients were evaluated in the clinic for AD severity and categorized using Clinical Dementia Rating (CDR) scale resulting in separation of patients into prodromal AD (CDR0.5) and advancing forms of AD dementia (mild-CDR1 and moderate/severe-CDR2/3). Separation of peripheral myeloid cells into mature monocytes or immature MDSCs permitted the delineation of population changes from flow cytometric analysis, RNA phenotype analysis, and functional studies using T cell suppression assays and monocyte suppression assays.

**Results:**

During stages of AD dementia (CDR1 and 2/3) peripheral myeloid cells increase their pro-inflammatory gene expression while at early stages of disease (prodromal AD—CDR0.5) pro-inflammatory gene expression is decreased. MDSCs are increased in prodromal AD compared with controls (16.81% vs 9.53%) and have markedly increased suppressive functions: 42.4% suppression of activated monocyte-produced IL-6 and 78.16% suppression of T cell proliferation. In AD dementia, MDSC populations are reduced with decreased suppression of monocyte IL-6 (5.22%) and T cell proliferation (37.61%); the reduced suppression coincides with increased pro-inflammatory signaling in AD dementia monocytes.

**Conclusions:**

Peripheral monocyte gene expression is pro-inflammatory throughout the course of AD, except at the earliest, prodromal stages when pro-inflammatory gene expression is suppressed. This monocyte biphasic response is associated with increased numbers and suppressive functions of MDSCs during the early stages and decreased numbers and suppressive functions in later stages of disease. Prolonging the early protective suppression and reversing the later loss of suppressive activity may offer a novel therapeutic strategy.

**Electronic supplementary material:**

The online version of this article (10.1186/s13024-018-0293-1) contains supplementary material, which is available to authorized users.

## Background

Alzheimer’s disease (AD) is the most common neurodegenerative disease and is characterized by cognitive impairment, amyloid-β (Aβ) deposition, and neurofibrillary tangle formation [1–5]. Increasing evidence suggests that immune mechanisms contribute to the pathogenesis of AD including reactive microgliosis in postmortem samples, increased microglial activation marker, translocator protein (TSPO), binding on positron emission tomography (PET), [6] and increased pro-inflammatory cytokines such as interleukin (IL)-6, IL-1β, TNF, and IFN-γ in the cerebrospinal fluid and serum [7–11]. Thus, neuroinflammation is potentially a target for immunomodulatory therapies [12, 13].

Genome-wide association studies implicate immune system dysfunction, particularly in myeloid-derived cells, as immune-related genes coding for triggering receptor expressed on myeloid cells 2 (TREM2) and CD33 confer increased risk for AD susceptibility [14–18]. Current studies focus on microglia, resident myeloid cells of the brain, throughout AD pathogenesis, but recent literature in neurodegenerative diseases suggest extensive neuro-immune cross-talk between the brain and peripheral immune system [19–22]. This cross-talk may derive either directly or indirectly from peripheral immune cells in the presence of a compromised blood brain barrier (BBB) such as in neurodegenerative disease [23–25]. Additionally during inflammatory insult and microglial depletion, peripheral macrophage engraftment into the CNS was observed with these cells retaining a distinct and lasting transcriptional and functional identity [26]. Thus peripheral immune myeloid cells could modulate disease progression and outcomes in the CNS. The accessibility of these peripheral myeloid cells, and the lack of accessibility to CNS microglia, prompted a detailed examination of blood monocyte populations during the pathoprogression of AD.

Immature and mature monocytes, here-after denoted as “peripheral myeloid cells,” originate from hematopoietic stem cells and mature into peripheral monocytes with the capability of differentiating into macrophages once they enter tissue parenchyma [27–31]. Changes and shifts in peripheral myeloid populations are indicators of disease onset and progression for a multitude of diseases; the pro-inflammatory phenotypes have direct effects on their specific disease [32–40]. A detailed analysis of peripheral monocyte population and phenotype changes have not been documented thoroughly in AD progression.

Myeloid-derived suppressor cells (MDSCs) are immature myeloid cells that exhibit robust suppressive function on T cell proliferation and mature myeloid cell function which has made them a target of multiple immunomodulatory therapies [41–44]. Chronic AD immune signaling provides a practical mechanism where MDSC activation and recruitment potentially promotes an immunosuppressive environment [45]. However, the actual role of MDSCs in AD pathology and progression remains unexplored. A detailed investigation into the role and function of mature and immature peripheral myeloid cells could provide insight into mechanisms of their direct involvement in AD pathogenesis as well as provide clues as to the changes in CNS myeloid cells through disease progression.

In this study, we investigated peripheral myeloid populations and their pro-inflammatory gene expression throughout the course of AD compared with age-matched controls. We also investigated changes in a novel and previously unrecognized cell type in AD, MDSCs, to determine their phenotypes during course of disease. Understanding the role of peripheral myeloid cells in AD-related inflammation and mechanisms of their immune suppression may lead to novel therapeutic interventions for the successful treatment of AD.

## Methods

### Patient recruitment and AD-defined population

AD patients and aged-matched healthy controls were recruited into the study from the Houston Methodist Nantz National Alzheimer’s Center (NNAC) based on National Institute on Aging-Alzheimer’s Association (NIA-AA) diagnostic criteria [46, 47]. The study was approved by the Houston Methodist IRB and all participants signed informed consent. Clinical evaluations for prodromal AD (CDR0.5) (*n* = 44), mild AD dementia (CDR1) (*n* = 25), moderate/severe AD dementia (CDR2/3) (*n* = 28), and age-matched, healthy controls (*n* = 54) were made under the direction of Dr. Joseph Masdeu and colleagues at the NNAC. Staging of dementia severity was based on the Clinical Dementia Rating (CDR) Scale (Table 1).

**Table 1:** Patient demographics

Diagnosis	CDR	Number	M,F	Age, yr. ± SD
Control	CDR 0	54	30, 24	69.81 ± 6.52
Prodromal AD	CDR0.5	44	21, 23	73.57 ± 6.57
Mild AD Dementia	CDR1	25	10, 15	74.48 ± 7.98
Moderate/Severe AD Dementia	CDR2/3	28	9, 19	71.29 ± 8.52

### Immune cell isolation

Immune cells were isolated from peripheral blood of participants using Lymphoprep density gradient (STEMCELL) followed by Human Pan Monocyte Isolation Kit (Miltenyi Biotec) for negative selection of human monocytes. Further isolation using anti-HLA-DR microbeads (Miltenyi Biotec) allowed for the separation of HLA-DR+, mature monocytes, and HLA-DR-, immature MDSCs (Additional file 1: Table S1**)**. T responder cells were isolated from the PBMC pool using CD4 + CD25+ regulatory T Cell Isolation Kit (Miltenyi Biotec) to obtain CD4 + CD25- cells.

### Flow cytometry

Fluorescent immune cell probes for the delineation of monocyte subsets included anti-human CD14-V450, anti-human CD16-FITC, anti-human HLA-DR-PerCP-Cy5.5, anti-human CD33-APC, anti-human CD11b-PE, and IgG isotype controls (ebioscience or BD Biosciences). Cells were counted using an LSRII flow cytometer with the data was analyzed with BD FACSDIVA software. The submitted flow cytometry gating paradigm (Additional file 2: Figure S2) depicts cell populations for analysis of mature monocyte populations and the immature MDSC population.

### RNA purification, RT-PCR analysis, and Nanostring

RNA was extracted from immune cell populations and in vitro cell experiments using Trizol reagent followed by Direct-zol RNA MiniPrep Kit (Zymo Research). Quantitative RT-PCR (qRT-PCR) experiments were performed using a One-Step RT-PCR kit with SYBR Green and run using Bio-Rad iQ5 Multicolor Real-Time PCR Detection Systems. Primers for the study were purchased from BioRad and the relative expression level of each mRNA was calculated using the ∆∆Ct method with normalization to β-actin and relative to control samples. RNA sample QC and Nanostring experiments were performed using Baylor College of Medicine Genomic and RNA Profiling Core. Monocyte RNA was run on the nCounter Human Inflammation Panel (Nanostring, Human v2). The panel consisted of 255 human inflammation-related genes with 15 internal reference genes. Analysis of data QC, normalization, and differential gene expression was performed using nSolver analysis software provided by the company. Normalized data counts generated from Nanostring experiment provided in Additional file 3: Figure S9.

### T cell suppression assays

Responder T cells and MDSCs were isolated using Miltenyi Biotec microbeads as previously described. CD4+ T responder cells were placed in a 96 well plate at a density of 50 k cells per well followed by co-culture of MDSCs at a ratio of 1:1, 1:1/2, and 1:1/4 (T cell: MDSC). CD3/CD28 T cell stimulation reagent is then added to the co-culture for five days followed by addition of tritium (Miltenyi). Proliferation measured via tritium incorporation.

### Myeloid cell suppression assays

Our lab has recapitulated protocols for the generation of mature monocyte cells from induced pluripotent stem cells (iPS cells). Yanagimachi et al. describes a detailed protocol and discussion of the process [48]. Isolation and polarization of CD14 cells into pro-inflammatory “M1” cells can be found in the culturing paradigm (Additional file 4: Figure S4). Briefly, iPSC-derived macrophages are grown from a control line and polarized to be pro-inflammatory. We then use MDSCs isolated from patients to co-culture with the pro-inflammatory iPSC-derived macrophages overnight (18 h) to look at suppressive function of the MDSCs on myeloid cells. We collected RNA from co-cultured pro-inflammatory macrophages for transcript analysis and cultured media for ELISA protein changes (Invitrogen).

## Results

### Peripheral myeloid cells have increased pro-inflammatory gene expression in AD dementia but significantly decreased expression in prodromal AD

**Fig. 1:**
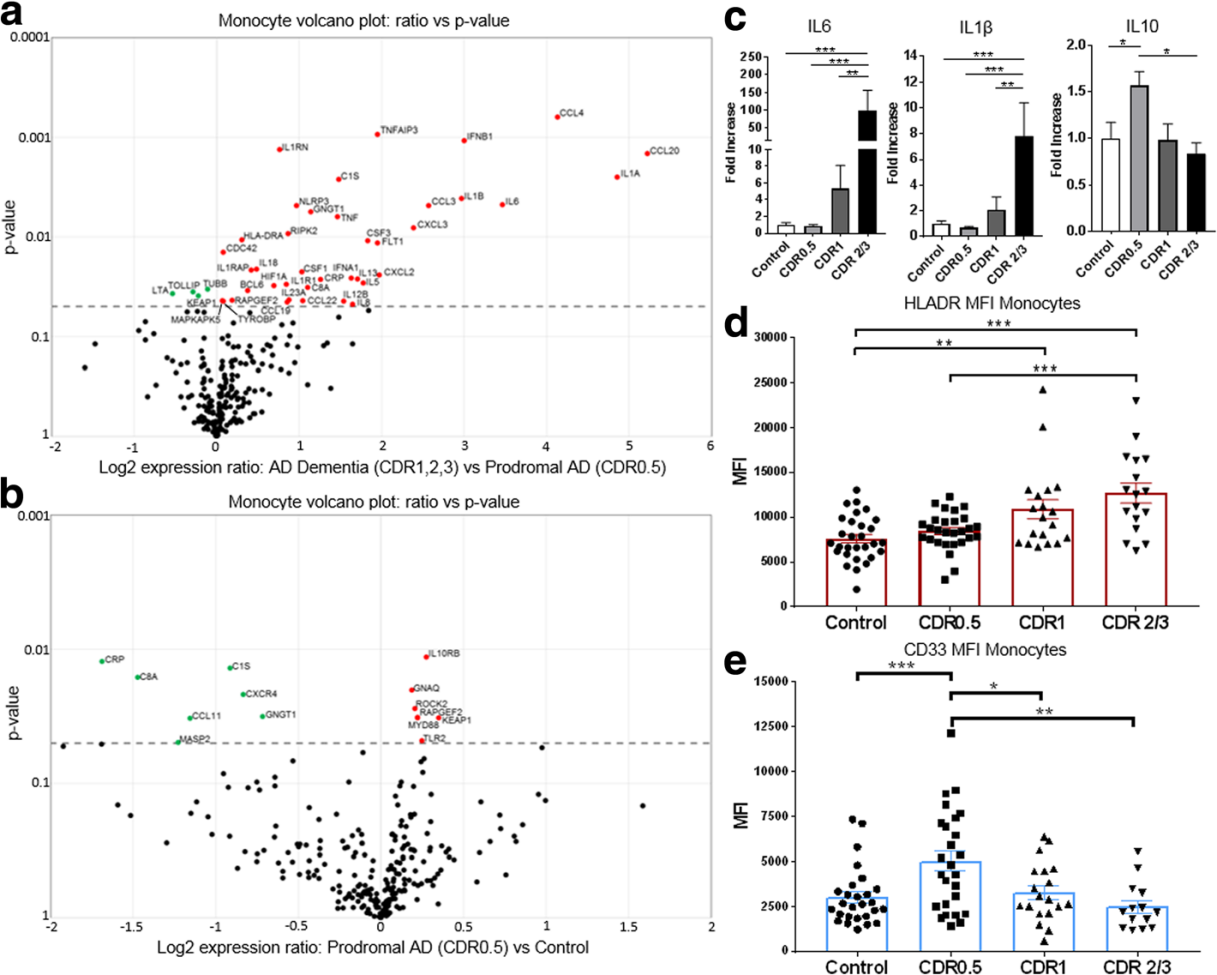
Peripheral myeloid cells are pro-inflammatory in AD dementia but suppressed/anti-inflammatory in prodromal AD. Volcano plots showing log2 expression ratios vs *p*-values of nCounter human inflammation v2 panel for (**a**) AD dementia (CDR1,2,3) vs prodromal AD (CDR0.5) and (**b**) Prodromal AD (CDR0.5) vs Control (AD Dementia, *n* = 10; Prodromal AD, *n* = 6; C *n* = 8). Analysis and statistics run using nSolver 3.0 analysis software. (**c**) Upregulation of pro-inflammatory gene expression of IL-6 and IL-1β in peripheral myeloid cells in CDR1 and 2/3 while IL-10 is increased in CDR0.5 peripheral myeloid cells (C *n* = 20, CDR0.5 n = 20, CDR1 *n* = 8, CDR2/3 *n* = 10). Numbers shown as averages ± SEM and with one-way ANOVA with Tukey’s post hoc test. **d** Flow cytometric median fluorescence intensity of pro-inflammatory HLADR is increased on the surface of CDR1 and CDR2/3 peripheral myeloid cells while (**e**) inhibitory motif, CD33, is increased on the surface of CDR0.5 peripheral myeloid cells and decreases with increasing AD burden. Numbers shown as averages ± SEM and with one-way ANOVA with Tukey’s post hoc test (C *n* = 30, CDR0.5 *n* = 27, CDR1 *n* = 17, CDR2/3 *n* = 13). *P*-values are *p < 0.05, **p < 0.01, and ***p < 0.001

RNA isolated from the blood-derived peripheral myeloid cells were analyzed using Nanostring chip and qRT-PCR to determine their inflammatory profiles. We detected increased pro-inflammatory RNA in AD dementia patients (CDR1, 2, 3) with increased expression of IL-6, IL-1β, NLRP3, TNF, IL-18, and HLA-DRA, among others. (Fig. 1a). There was little to no increase in inflammation-associated RNA signatures between controls and prodromal AD (CDR0.5) patient cells (Fig. 1b). In fact, there were decreased RNA levels of C-reactive protein (CRP), CXCR4, CCL11, and complement system components (C1s and C8a) in prodromal AD cells compared with controls. Follow-up analysis from additional patients using qRT-PCR corroborated the Nanostring data showing decreased expression of IL-6 and IL-1β in CDR0.5 peripheral myeloid cells but increased expression of these two cytokines in CDR1 and CDR2/3 peripheral myeloid cells (Fig. 1c). Additionally, anti-inflammatory IL-10 expression, as determined by qRT-PCR, was increased in CDR0.5 peripheral myeloid cells compared with controls but not in samples from CDR1 or CDR2/3 patients (Fig. 1c).

Surface level expression of HLA-DR, up-regulated during immune cell activation to promote pro-inflammatory signaling, was increased on mature monocytes from CDR1 and CDR2/3 compared with both controls and CDR0.5 (Fig. 1d). CD33 expression which is known to inhibit activation and cytokine release from myeloid cells was increased on cells from CDR0.5 patient monocytes but not on cells from controls and CDR1 and CDR2/3 patients (Fig. 1e**).** Gender and age of the controls and patients were not confounding variables in these experiments (Additional file 5: Figure S3 and Additional file 6: Figure S6).

### Alterations in mature and immature monocyte populations through AD progression

**Fig. 2:**
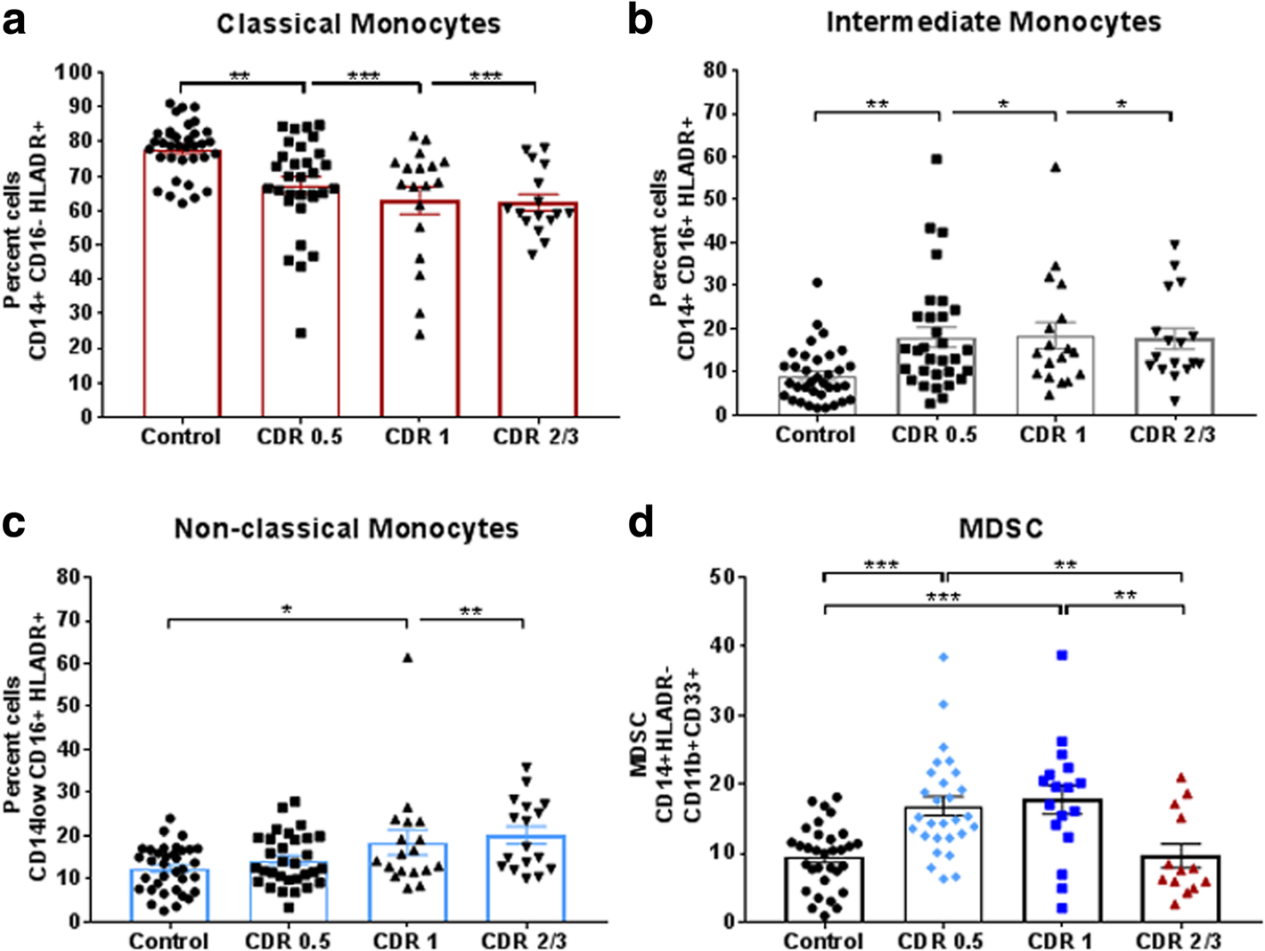
Monocyte populations shift as AD burden increases. Monocyte population changes via flow cytometric analysis of CD14 and CD16 expression on HLADR+ myeloid immune cells. Decreases in (**a**) classical monocytes (CD14+ CD16-) with increasing disease burden with a redistribution of monocytes into (**b**) intermediate (CD14+ CD16+) and (**c**) non-classical populations (CD14low CD16+). Numbers shown as averages ± SEM and with one-way ANOVA with Tukey’s post hoc analysis (Control *n* = 35, CDR0.5 *n* = 31, CDR1 *n* = 18, CDR2/3 *n* = 17). Increased MDSC populations (**d**) in CDR0.5 and CDR1 patients compared with controls as percent of total CD14 cell population. MDSC numbers fall to control levels in CDR2/3 patients (Control *n* = 32, CDR0.5 *n* = 28, CDR1 *n* = 17, CDR2/3 *n* = 13). Numbers shown as averages ± SEM and with one-way ANOVA with Tukey’s post hoc test. *P*-values are *p < 0.05, **p < 0.01, and ***p < 0.001

We observed a progressive decline of the classical monocytes (CD14^+^CD16^−^) with increasing AD burden (Fig. 2a) leading to a redistribution of the populations to intermediate (CD14^+^CD16^+^) and non-classical populations (CD14^low^CD16^+^). Intermediate populations in CDR0.5, 1, and 2/3 were increased compared with controls (Fig. 2b). CDR1 and CDR2/3 non-classical populations were increased compared with controls (Fig. 2c**).** Using CD14^+^, HLA-DR^−^, CD11b^+^, and CD33^+^ as cell surface marker for MDSCs, there was an increased number of MDSCs in CDR0.5 (16.81%) and CDR1 (17.74%) patients compared with age-matched controls (9.53%). CDR2/3 MDSC levels (9.64%) were similar to control levels (Fig. 2d). Gender and age did not confound monocyte population changes (Additional file 7: Figure S5).

### Pro-inflammatory responses of monocytes from AD dementia patients and anti-inflammatory responses of MDSCs from prodromal AD patients

**Fig. 3:**
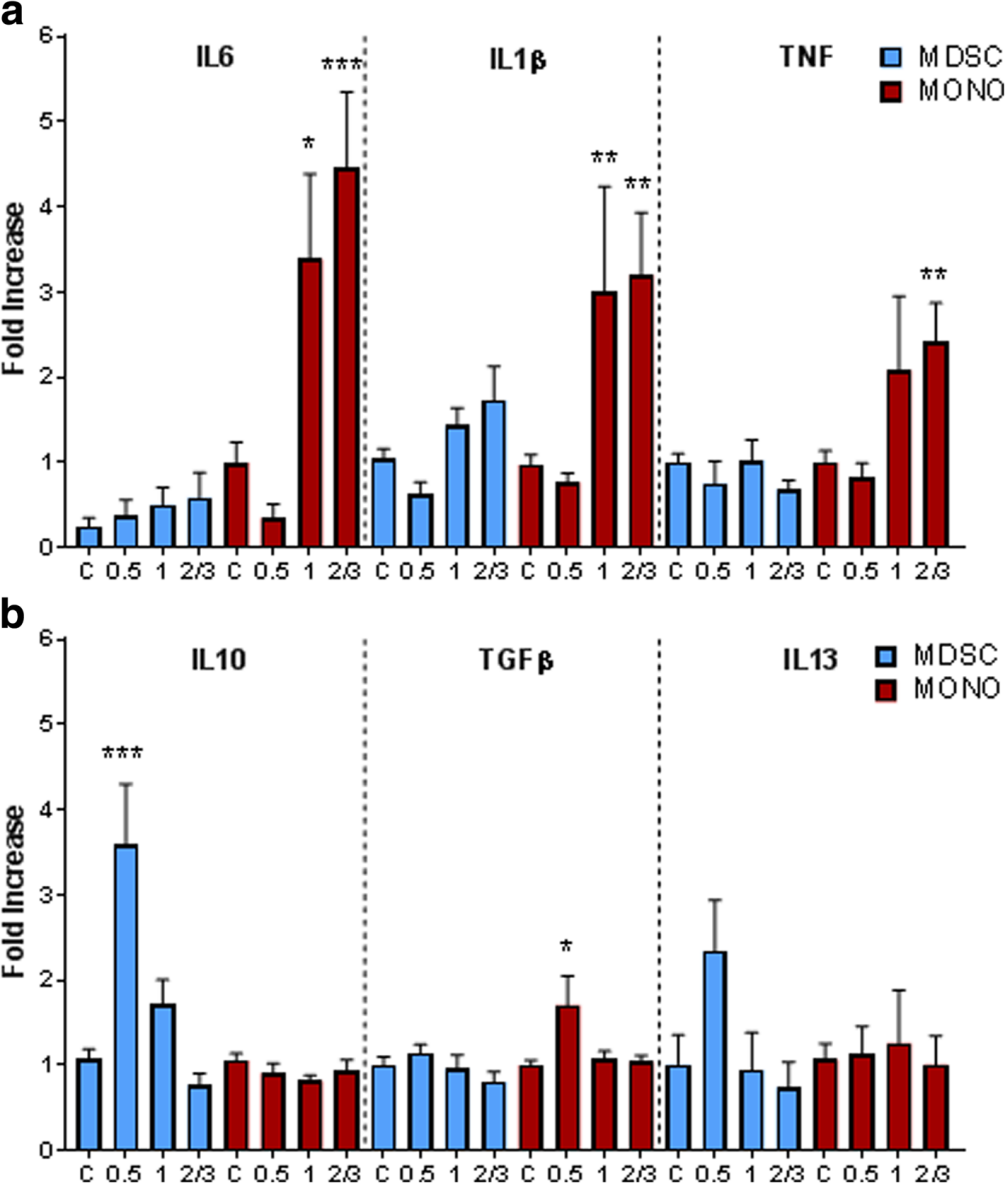
Mature monocytes are pro-inflammatory in AD dementia while MDSCs are anti-inflammatory in prodromal AD. Isolated mature monocytes and MDSCs displayed differential inflammatory gene expression profiles. **a** Mature monocytes from CDR1 and CDR2/3 display increased pro-inflammatory cytokine expression via IL-6, IL-1β, and TNF. **b** MDSCs from CDR0.5 patients have increased anti-inflammatory IL-10 and IL-13 expression. TGFβ transcripts were increased in CDR0.5 monocytes. One-way ANOVA with Tukey’s post hoc analysis (Control *n* = 13–14, CDR0.5 *n* = 8, CDR1 *n* = 7, CDR2/3 *n* = 10). P-values are *p < 0.05, **p < 0.01, and ***p < 0.001

Following separation of MDSCs from monocytes, RNA analysis demonstrated increases in pro-inflammatory signaling in monocytes from AD dementia patients. Specifically, there were increased mRNA expression of IL-6, IL-1β, and TNF in mature monocytes isolated from CDR1 and CDR2/3 patients when compared with controls (Fig. 3a). Additionally, there was a trend for a decrease in IL-6 transcript from CDR0.5 patient monocytes while the IL-1β and TNF were not different than controls. In evaluating anti-inflammatory signaling in MDSC populations, IL-10 and IL-13 transcripts were increased in CDR0.5 but then decreased as the disease progressed to CDR1 and CDR2/3 (Fig. 3b). Interestingly, anti-inflammatory TGF-β was increased in mature monocytes from CDR0.5 patients but not from the monocytes of later stages.

### Pro-inflammatory myeloid cells and T cell proliferation are suppressed by prodromal AD MDSCs but not AD dementia MDSCs

**Fig. 4:**
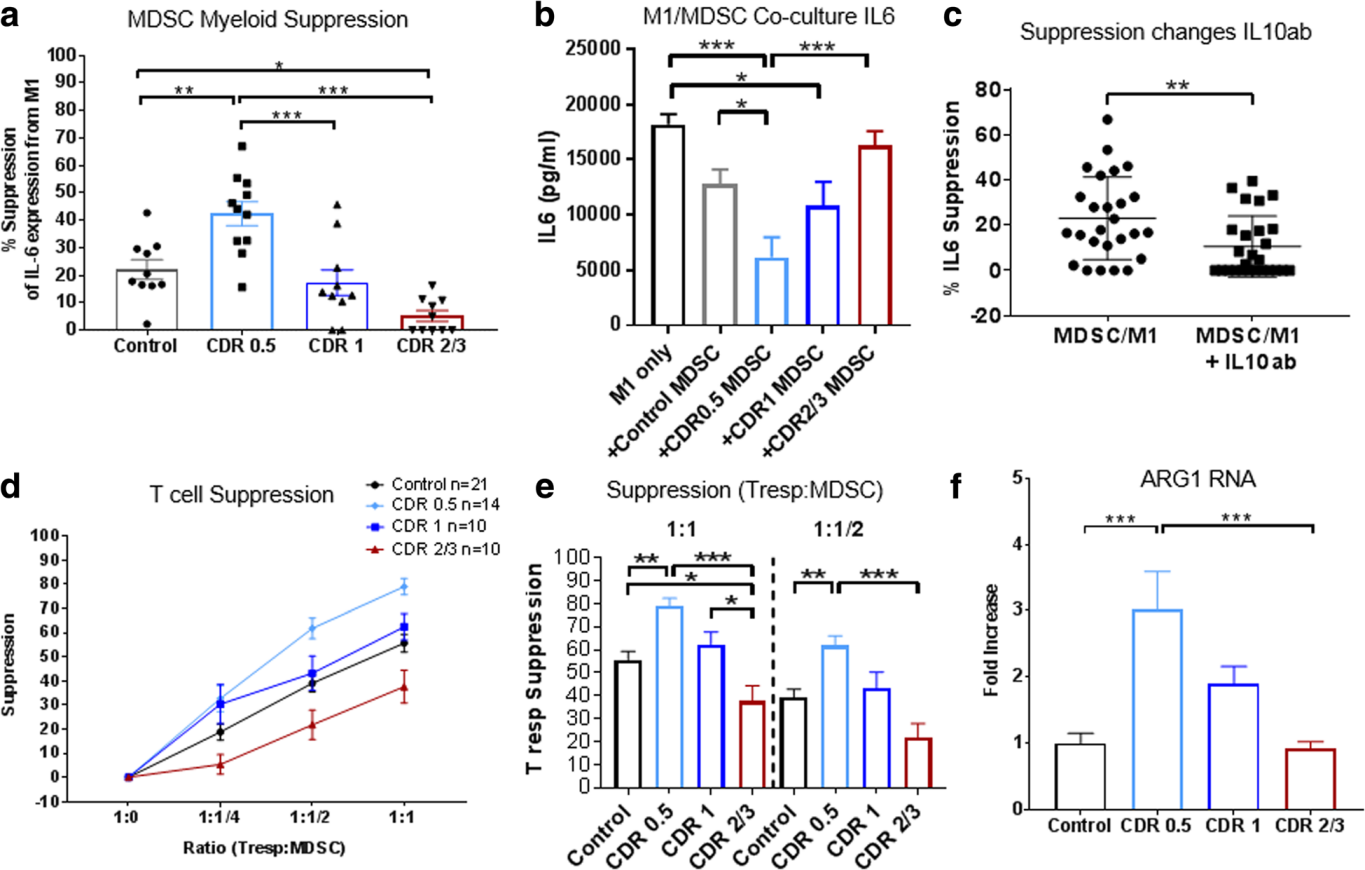
Stage-specific MDSC suppression of pro-inflammatory myeloid cells and T cell proliferation. Isolated patient MDSCs are co-cultured overnight (18 h) at 1:1 ratio with iPSC-generated M1 pro-inflammatory macrophages. **a** Production of IL-6 transcripts were highly suppressed by MDSCs from CDR0.5 patients while that suppression decreases as the patient advances to CDR1 and CDR2/3. (Control *n* = 10, CDR0.5 *n* = 11, CDR1 *n* = 10, and CDR2/3 *n* = 10). **b** Conditioned media from iPSC-M1 and patient MDSC co-cultures were analyzed via ELISA for changes in IL-6 protein. Changes in protein levels mirrored the IL-6 RNA suppression with CDR0.5 MDSCs causing a decrease in IL-6 protein production by M1 cells. These suppressive effects tapered off with advancing disease condition (Control *n* = 6, CDR0.5 *n* = 6, CDR1 *n* = 4, and CDR2/3 *n* = 5). **c** Addition of 5μg/mL neutralizing antibody for IL-10 reduces the IL-6 suppression in our MDSC: M1 assays. Numbers shown as averages ± SEM and statistics utilized were one-way ANOVA with Tukey post hoc (**a**, **b**) and student’s t-test (**c**). Isolated MDSCs were co-cultured with T responder cells at ratios of 1:1/4, 1:1/2, and 1:1 and stimulated for proliferation. **d**, **e** CDR0.5 MDSCs show hypersuppression of T cell proliferation compared with other groups. Late CDR2/3 MDSCs lost their T cell proliferation suppression below that of controls (Control *n* = 21, CDR0.5 *n* = 14, CDR1 *n* = 10, and CDR2/3 *n* = 10). **f** Arg1 expression in isolated MDSCs increase in CDR0.5 but decrease with increasing AD burden (Control *n* = 12, CDR0.5 *n* = 8, CDR1 *n* = 8, CDR2/3 *n* = 10). Numbers shown as averages ± SEM with one-way ANOVA with Tukey’s post hoc analysis. *P*-values are *p < 0.05, **p < 0.01, and ***p < 0.001

We investigated the suppressive capacity of MDSCs on a pro-inflammatory macrophage population. We co-cultured patient-isolated MDSCs with pro-inflammatory iPSC-derived macrophages overnight to examine attenuation of pro-inflammatory IL-6 transcript and protein. MDSCs from CDR0.5 patients attenuated IL-6 transcript expression (42.4%) compared with control MDSCs (22.09%) (Fig. 4a). This attenuation of IL-6 expression decreased in CDR1 (17.33%) and CDR2/3 (5.22%) patients. In examination of the co-cultured media, we find decreased IL-6 protein when we co-culture pro-inflammatory macrophages (alone-18,208 pg/mL IL-6) with CDR0.5 MDSCs (6154 pg/mL). This suppressive effect decreases as patients advance to CDR1 (10,856 pg/mL) and later into CDR 2/3 (16,348 pg/mL). Control MDSCs in our experiment were able to reduce pro-inflammatory IL-6 output to 12,791 pg/mL while MDSCs alone did not produce IL-6 protein. Gender and age did not affect suppressive capacity of control or patient MDSCs (Additional file 8: Figure S7). Our RNA expression data from isolated mature monocytes and MDSCs displayed increased IL-10 transcript from CDR0.5 MDSCs. Adding an IL-10 neutralizing antibody (IL10ab) to the MDSC: M1 cultures at 5μg/mL significantly reduced suppression of IL-6 transcripts (Fig. 4c**),** suggesting that secretion of IL-10 contributes to the MDSC-mediated suppression.

The suppression profiles of MDSCs were next examined when co-cultured with CD4^+^ Tresp. CDR0.5 and CDR1 MDSCs showed increased suppression of autologous Tresp proliferation compared with the suppression of control MDSCs. Similar to the decreased numbers of MDSCs, there was decreased MDSC suppressive function in CDR2/3 compared with all groups. At a 1:1 ratio (Tresp: MDSC), CDR0.5 and CDR1 yielded 79.03% and 61.81% suppression, respectively, compared with control 55.7% suppression while CDR2/3 MDSCs dropped to 37.61% (Fig. 4d, e). When examining the expression of arginase-1 (Arg1) as a mechanism to block T cell proliferation, Arg1 expression was increased in CDR0.5 MDSCs compared with controls and subsequently decreased in CDR1 and then in CDR2/3 patients (Fig. 4f). T cell suppression by MDSCs positively correlated with myeloid cell suppression; patients with early disease possess increased suppressive function while suppressive function is progressively impaired as disease advances (Additional file 9: Figure S8).

## Discussion

The analyses of peripheral myeloid cells in AD patients provided three important findings. First, pro-inflammatory gene-expressing phenotypes of peripheral monocytes are increased in CDR1,2,3 patients. Second, pro-inflammatory gene-expressing phenotypes of peripheral monocytes are decreased in CDR0.5 patients. Third, MDSCs are increased in number and suppressive function in CDR0.5 and decreased in CDR2/3. MDSC from CDR0.5 suppressed Tresp proliferation and iPSC-derived pro-inflammatory myeloid cells in vitro more effectively than control MDSCs. MDSCs from CDR2/3 patients had significantly decreased suppressive function for both Tresp proliferation and pro-inflammatory myeloid cells.

Activation of myeloid cells has been extensively studied in humans and animal models of neurodegeneration, including AD, with much of the focus on brain microglia which are difficult to study directly during the course of disease. Peripheral myeloid cells have similar functions to brain microglia including chemotaxis, phagocytosis, and cytokine expression but constitute a cell population that can be collected and studied through simple, minimally-invasive methods [28, 49–51]. Activation of immune cells can be assessed using flow cytometry by analyzing HLA-DR, a MHC class II cell surface receptor that is upregulated upon antigen presenting cell activation. HLA-DR expression was increased on mature monocytes from CDR1 and CDR2/3 patients when compared with controls and CDR0.5 which matches the pro-inflammatory gene expression seen in these patients. CDR0.5 monocytes did not exhibit pro-inflammatory gene expression but displayed increased inhibitory CD33 cell surface expression compared with controls and this expression diminished in CDR1 patients while decreasing below control values in CDR2/3 patients. While some studies implicate differential activation and polarization of immune cells due to the age and gender of the patient, we did not observe any significant effects contributed by these factors in our control or AD datasets. Advanced studies into gender and age-specific contributions to neurodegenerative disease are warranted.

The peripheral pattern of immune cell deactivation and reactivation is in accord with the biphasic immune marker profile reported in a study of CSF immune makers in AD [52]; decreases in immune signaling were noted early in the course of disease and a strong increase in immune signaling was noted in later stages of disease. Additionally, a longitudinal imaging study of AD patients found initial microglial activation upon diagnosis followed by a reduction of activation as patients went through mild cognitive impairment stages. As patients progressed into later stages of AD, microglial activation increased again suggesting that there might be two activation stages; the first being a protective activation state while the later peak being a more pro-inflammatory and destructive process [53].

Many studies utilize changes and shifts in monocyte populations to identify and predict inflammatory disease and progression in a multitude of inflammation-based disorders [38–40]. Previous AD studies suggest monocyte involvement in AD although our analysis showed no difference of total monocyte number between controls and through the progression of AD [54–57]. With no studies focused on AD monocyte population shifts, our results present a progressive decline of the classical monocyte population (CD14^+^CD16^−^) with a subsequent increase in both the intermediate (CD14^+^CD16^+^) and non-classical (CD14^low^CD16^+^) subsets as disease progresses. Shifting of populations from the classical population, a patrolling subset, into intermediate and non-classical populations reflects monocyte maturation and pro-inflammatory activation [58]. Identification of mechanisms whereby patient monocytes are shifting to their subsets and how they are inhibited early while being activated with increasing disease burden is needed.

MDSCs are immature myeloid cells that expand under pathological conditions such as cancer and immune disorders but have yet to be fully characterized in patients or animal models of AD. These cells suppress pro-inflammatory immune cells including mature myeloid cells and CD4^+^/ CD8^+^ T cells. MDSCs are roughly divided into early-stage, granulocytic, and monocytic MDSCs with the latter being most suppressive and characterized as CD14^+^/HLA-DR^−^/CD11b^+^/CD33^+^ cells [41, 59]. MDSC numbers were increased in CDR0.5 and CDR1 patients, and decreased in CDR2/3 patients. While monocytic MDSCs are undocumented in AD, a previous study documented an increase in granulocytic MDSCs in amnestic mild cognitive impaired patients compared with mild AD patients. However, this previous study did not provide functional data, thus it is not known whether granulocytic MDSCs have differential functions as the course of AD progresses or if AD granulocytic MDSCs have different properties compared to those of healthy controls [60].

MDSCs and mature pro-inflammatory monocytes have opposing roles in inflammation. The former provides strong immunosuppression while the latter contributes to pro-inflammatory signaling. Analyses of these phenotypes and signaling mechanisms in advancing AD would provide important information on the state of the immune system during the progression of AD. Additionally, these cells may directly engraft into the CNS given the evidence of a compromised BBB based upon the leakage of blood-derived constituents in postmortem AD brains [61–63], the increased CSF/serum or CSF/plasma ratios of albumin [64–66], and imaging showing an age-dependent increase in BBB permeability in the hippocampus, dentate gyrus, and CA1 region [67]. Additionally, dysregulation of the neurovascular unit, responsible for maintaining BBB integrity, is known to accompany AD pathology [68]. Peripheral immune cell involvement and engraftment into the CNS are postulated to play important roles in AD [69, 70]. With that said, roles of infiltrating cells during CNS disease pathogenesis is controversial, contextual, and disease/stage-dependent [22, 56, 71–73]. Whether and how peripheral myeloid cells dictate progression of AD necessitates further investigation, especially the question of peripheral immune cell migration into the CNS and other mechanisms of neuro-immune cross-talk.

Microglia and monocytes, through genetics and activation status in AD, play a critical role in AD pathogenesis [6, 14–18]. Their responsibilities include phagocytosis, cytokine secretion, and restoration of homeostatic conditions with impairments to these functions being deleterious to sensitive neuronal environments in AD. We have developed CD14 myeloid cells from control iPS cells (unpublished Zhao et al.) and polarized them to be pro-inflammatory to examine differential effects of control/AD MDSCs on activated myeloid cells. Pro-inflammatory polarization of iPS cells provides a consistent, activated cell type to compare suppression between patients without the confounding variable of primed or differentially activated mature monocytes from patients.

MDSCs from CDR0.5 patients were able to suppress pro-inflammatory IL-6 expression and protein from activated cells more than controls while CDR1 and CDR2/3 markedly lost their suppressive function. IL-6 transcript and protein analysis were used due to MDSCs producing no IL-6 while IL-6 is a predominant pro-inflammatory output from activated myeloid cells [74]. The IL-10 data from the RNA studies provided a mechanistic target for MDSC suppression of the myeloid cells. Adding IL-10ab to co-cultures reduced MDSC suppression of myeloid IL-6 transcripts that was most pronounced with CDR0.5 co-cultures but also observed in the other groups. IL-10ab only partially blocked IL-6 suppression leaving other additional mechanisms contributing to suppression, either through soluble signaling or direct cell contact. An extensive study into the intrinsic differences between MDSCs from prodromal and AD dementia is warranted to comprehensively parse out the predominating mechanisms of their suppressive functions and which of these are lost through AD progression.

Co-culture of MDSCs with Tresps showed increased CDR0.5 and CDR1 MDSC suppression compared with controls. The mechanism for MDSC suppression of T cell proliferation is through metabolism of arginine via increased Arg1 transcript in MDSCs [75, 76]. Arg1 gene expression increased in CDR0.5 MDSCs and then diminished as AD advances. Analysis of CDR1 MDSC suppression shows enhanced T cell proliferation but decreased M1 suppression which may suggest a suppressive function transition occurring at this stage.

Limitations of these studies involve lack of direct evidence that peripheral myeloid cells enter the CNS parenchyma and influence disease progression in AD. Nevertheless, the definitive evidence for alterations in the BBB in AD, the comparability of CSF findings and peripheral myeloid cell phenotype alterations, and the PET scan evidence of increasing neuroinflammation during the course of disease are all in accord with extensive neuro-immune cross-talk between the brain and peripheral immune system. Advances in neuroimaging with specialized ligands may provide tools for analyzing central vs peripheral identities as well as the anti vs pro-inflammatory phenotypes contributing to AD pathogenesis. The identification of distinct transcriptional and functional phenotypes could assist in developing these technologies [26]. Additionally, complex networks and signaling cascades involving additional cells outside our examination might assist in dictating inflammatory environments and cell phenotypes. Examination of these cells, both individually and in concert with each other, will help establish a more definitive immune environment during AD and through its course.

## Conclusions

This study documents that peripheral monocytes are pro-inflammatory in advancing stages of AD but not in prodromal AD. The pro-inflammatory responses of monocytes from prodromal AD patients are suppressed while advancing AD patients monocytes lose this suppression, and become activated and pro-inflammatory. Numbers and suppressive functions of MDSCs are increased in prodromal AD and decreased in patients with advancing AD and correlate with pro-inflammatory expression of AD monocytes. MDSCs also suppress Tresp which can readily enter the CNS, and loss of T effector suppression can significantly enhance inflammatory disease pathology. These findings provide a novel inflammatory paradigm that may have confounded early therapeutic interventions and provides a new basis for how future studies and treatments should be designed. Additionally, we have documented the significant impact of AD MDSCs on immune cell subsets. Understanding the role of early enhanced immunosuppression in prodromal AD and the subsequent dysfunction of this process in AD dementia may lead to novel therapeutic strategies.

## Additional files

Additional file 1: **Table S1.** Cell Populations. (TIF 58 kb)

Additional file 2: **Figure S2.** Flow cytometry gating schemes for monocyte and MDSC populations. (TIF 539 kb)

Additional file 3: **Figure S9.** Nanostring dataset for RNA analysis of CDR rated patients and age-matched controls. (XLSX 65 kb)

Additional file 4: **Figure S4.** iPSC-derived M1 and MDSC co-culture paradigm. (TIF 231 kb)

Additional file 5: **Figure S3.** (a) Correlation data of inflammatory RNA expression and subject age from peripheral myeloid cells. Data represent analyses of RNA expression with controls only, patients with varying levels of AD, and all groups combined (Control *n* = 20, AD *n* = 38). (b) Analyses of gender contributions to pro-inflammatory RNA expression among controls, CDR0.5, CDR1, and CDR2/3 (Control *n* = 10/10 M/F, CDR0.5 *n* = 6/14 M/F, CDR1 *n* = 5/3 M/F, CDR2/3 *n* = 4/6 M/F). Graphs show average ± SEM with statistics run using two-way ANOVA with Sidak’s multiple comparisons test. No statistical difference observed after age and gender data stratification unless signified by *p < 0.05, **p < 0.01, or ***p < 0.001. (TIF 156 kb)

Additional file 6: **Figure S6.** (a) Correlation data between age and protein expression of HLADR and CD33 analyzed via flow cytometry (Control *n* = 30, AD *n* = 57). (b) Analyses of gender contributions to HLADR and CD33 expression on mature myeloid cells isolated from controls, CDR0.5, CDR1, and CDR2/3 (Control *n* = 14/16 M/F, CDR0.5 *n* = 13/14 M/F, CDR1 *n* = 8/10 M/F, CDR2/3 *n* = 3/10 M/F). Graphs show average ± SEM with statistics run using two-way ANOVA with Sidak’s multiple comparisons test. No statistical difference observed after age and gender data stratification unless signified by *p < 0.05, **p < 0.01, or ***p < 0.001. (TIF 194 kb)

Additional file 7: **Figure S5.** (a) Correlation data between age and monocyte population changes. Analyses performed examined the ages of controls, varying levels of AD, and combined groups for correlations in changes in classical monocytes, intermediate monocytes, non-classical monocytes, and MDSCs (Control *n* = 35, AD *n* = 66). (b) Analyses of gender contributions to monocyte population changes among controls, CDR0.5, CDR1, and CDR2/3 (Control n = 20/15 M/F, CDR0.5 *n* = 15/16 M/F, CDR1 *n* = 8/10 M/F, CDR2/3 *n* = 5/12 M/F). Graphs show average ± SEM with statistics run using two-way ANOVA with Sidak’s multiple comparisons test. No statistical difference observed after age and gender data stratification unless signified by *p < 0.05, **p < 0.01, or ***p < 0.001. (TIF 246 kb)

Additional file 8: **Figure S7.** (a) Analysis of gender contribution to MDSC suppressive function on pro-inflammatory M1 cells (Control *n* = 6/4 M/F, CDR0.5 n = 5/6 M/F, CDR1 *n* = 4/6 M/F, CDR2/3 *n* = 3/7 M/F). Graph shows average ± SEM with statistics run using two-way ANOVA with Sidak’s multiple comparisons test. No statistical difference observed after gender data stratification unless signified by *p < 0.05, **p < 0.01, or ***p < 0.001. (TIF 25 kb)

Additional file 9: **Figure S8.** (a) Correlation plot graphing T resp. proliferation suppression and myeloid IL-6 transcript suppression at 1:1 ratio of responding cells to MDSCs (*R* = .7288 *p* = 0.004). (b) IL-6 control experiment whereby MDSCs from controls (*n* = 6) and AD patients from various stages (*n* = 12) do not express IL-6 transcript when cultured alone in LPS/IFNγ treatments. Corroboration with no IL-6 protein in the MDSC only treated media when analyzed via ELISA (data not shown). (TIF 29 kb)
