# Supplementary figures and images for: Functional alterations of myeloid cells during the course of Alzheimer’s disease
Source: Mol Neurodegener. 2018 Nov 13;13:61. doi: 10.1186/s13024-018-0293-1 (PMC6233576; doi:10.1186/s13024-018-0293-1)

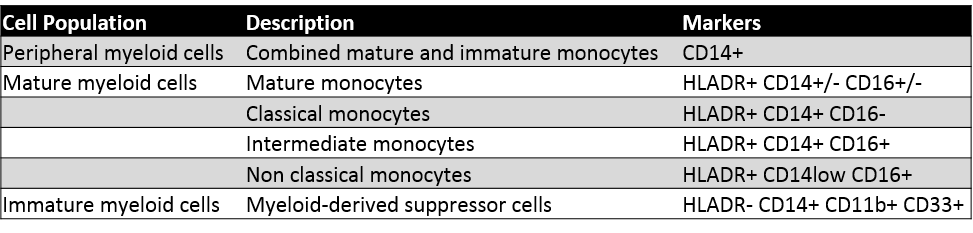

Supplement: Supplementary file 1 — Table S1. Cell Populations. (TIF 58 kb) [file 13024_2018_293_MOESM1_ESM.tif]

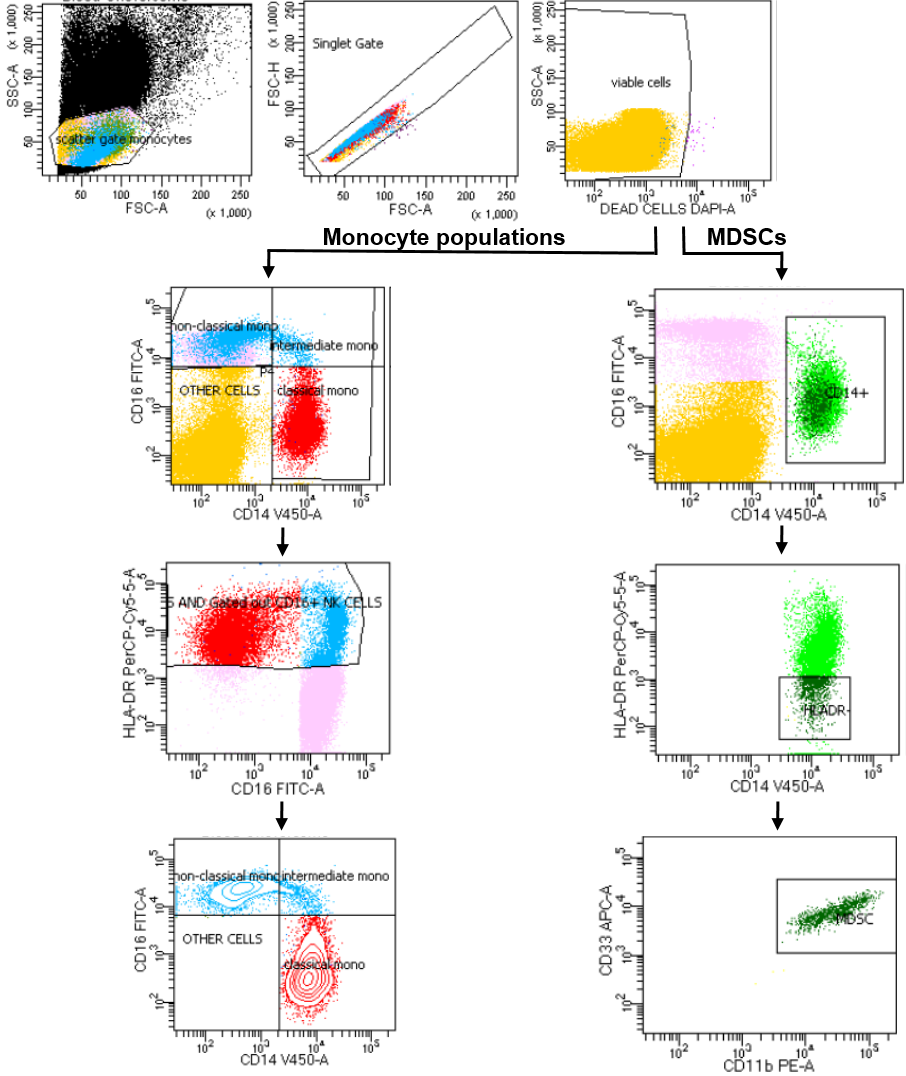

Supplement: Supplementary file 2 — Figure S2. Flow cytometry gating schemes for monocyte and MDSC populations. (TIF 539 kb) [file 13024_2018_293_MOESM2_ESM.tif]

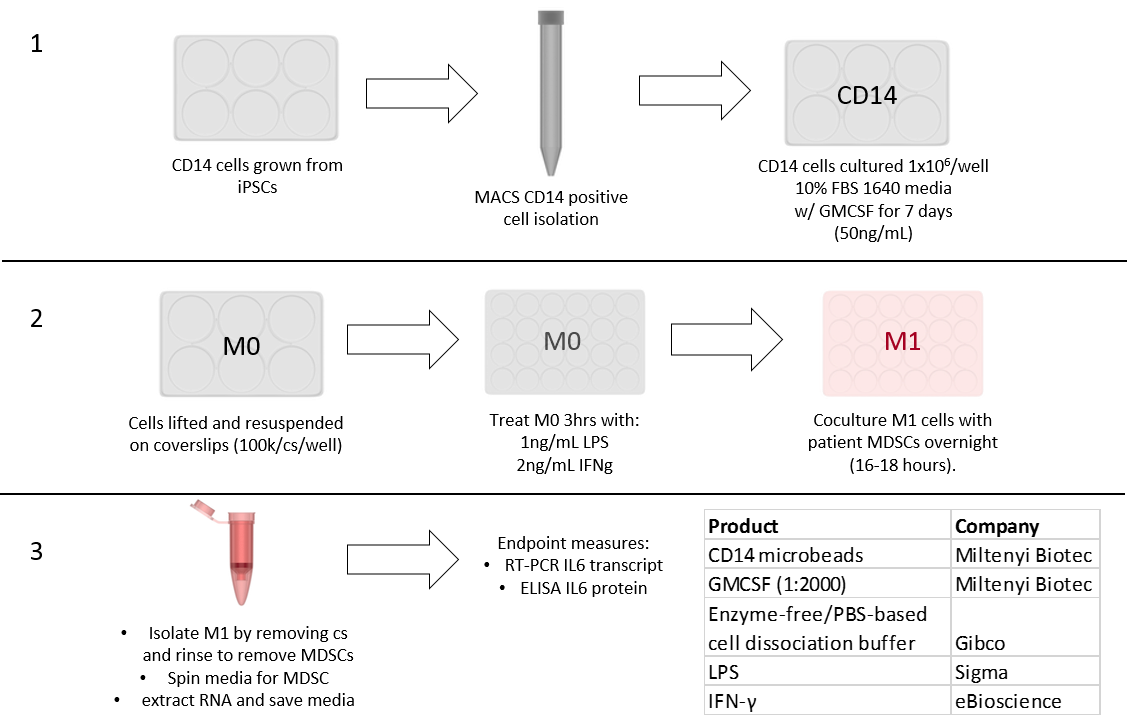

Supplement: Supplementary file 4 — Figure S4. iPSC-derived M1 and MDSC co-culture paradigm. (TIF 231 kb) [file 13024_2018_293_MOESM4_ESM.tif]

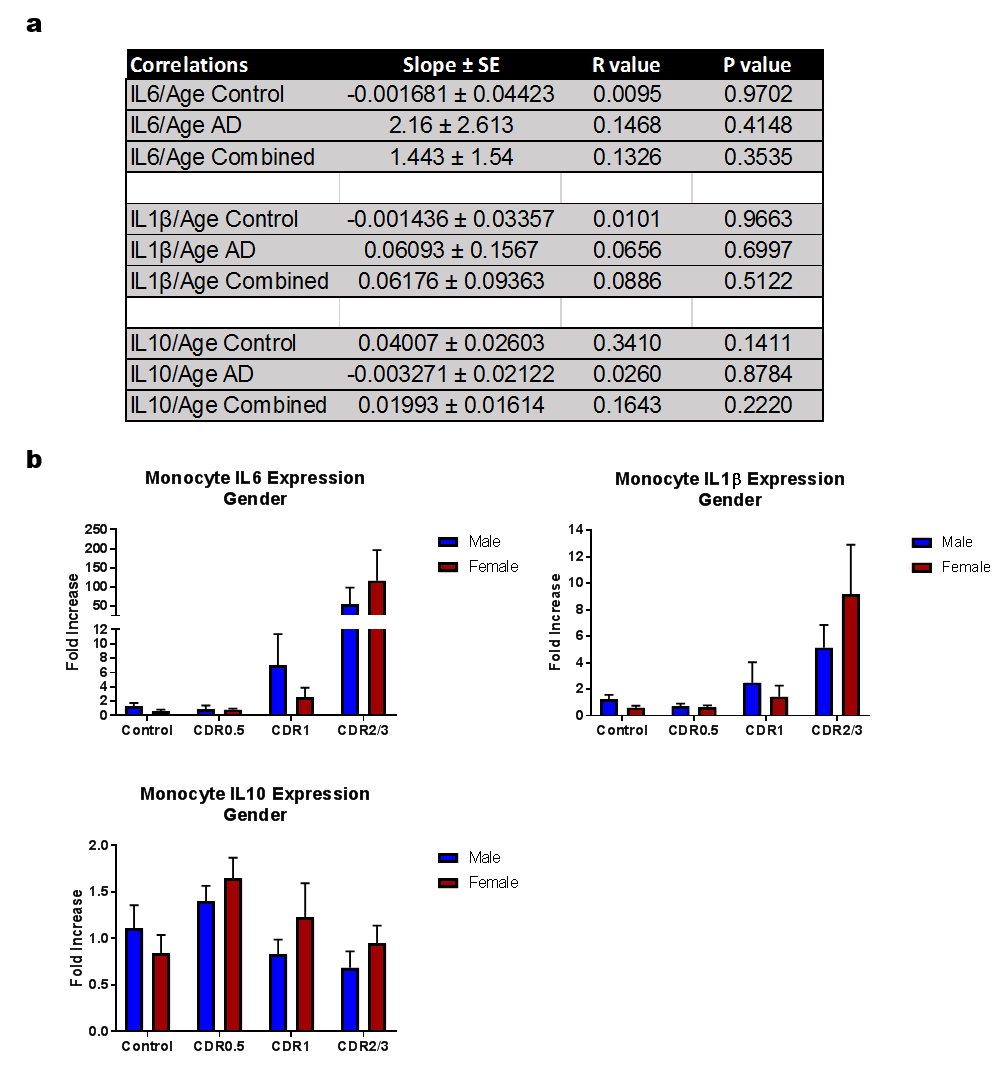

Supplement: Supplementary file 5 — Figure S3. (a) Correlation data of inflammatory RNA expression and subject age from peripheral myeloid cells. Data represent analyses of RNA expression with controls only, patients with varying levels of AD, and all groups combined (Control n = 20, AD n = 38). (b) Analyses of gender contributions to pro-inflammatory RNA expression among controls, CDR0.5, CDR1, and CDR2/3 (Control n = 10/10 M/F, CDR0.5 n = 6/14 M/F, CDR1 n = 5/3 M/F, CDR2/3 n = 4/6 M/F). Graphs show average ± SEM with statistics run using two-way ANOVA with Sidak’s multiple comparisons test. No statistical difference observed after age and gender data stratification unless signified by *p < 0.05, **p < 0.01, or ***p < 0.001. (TIF 156 kb) [file 13024_2018_293_MOESM5_ESM.tif]

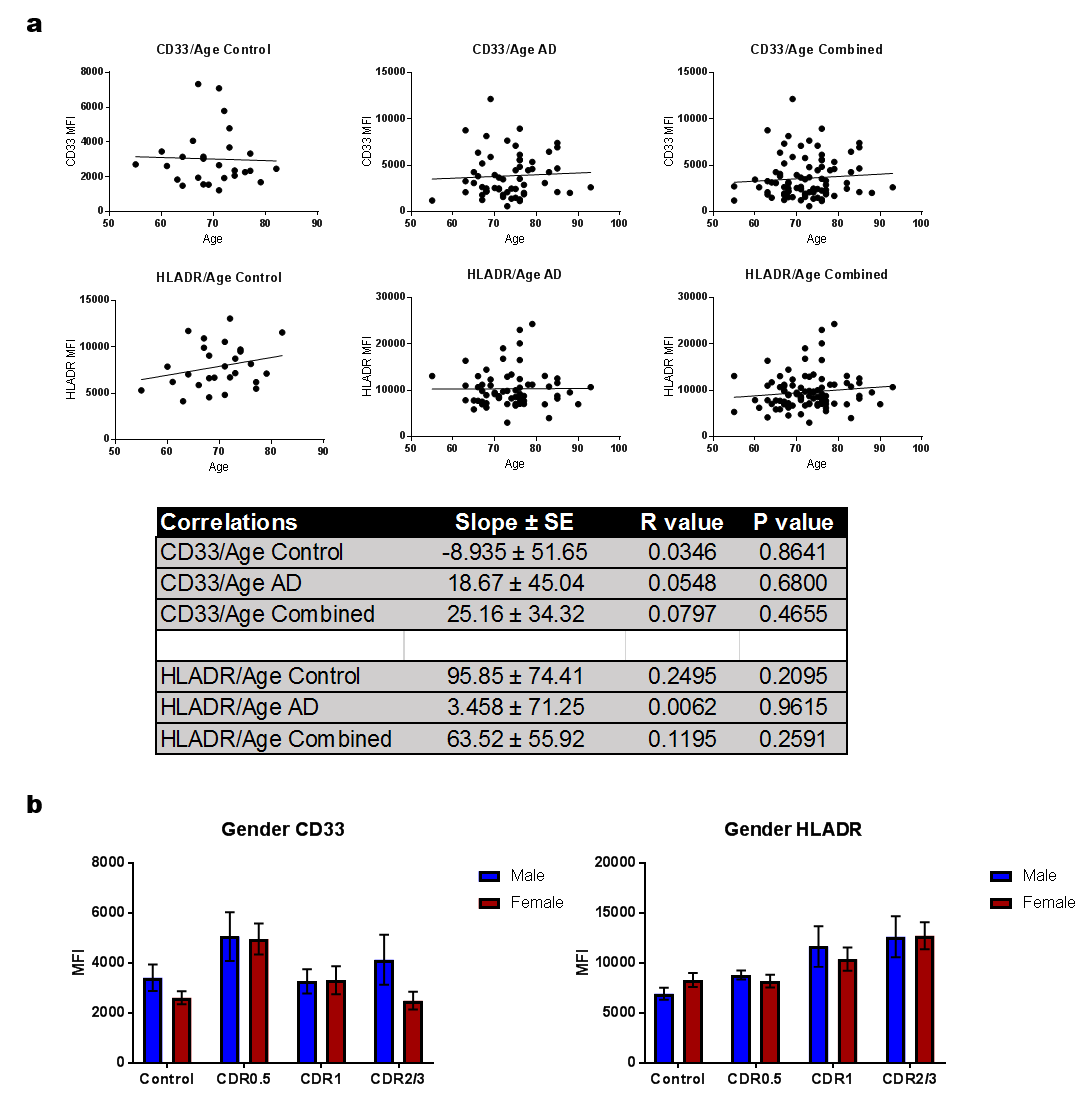

Supplement: Supplementary file 6 — Figure S6. (a) Correlation data between age and protein expression of HLADR and CD33 analyzed via flow cytometry (Control n = 30, AD n = 57). (b) Analyses of gender contributions to HLADR and CD33 expression on mature myeloid cells isolated from controls, CDR0.5, CDR1, and CDR2/3 (Control n = 14/16 M/F, CDR0.5 n = 13/14 M/F, CDR1 n = 8/10 M/F, CDR2/3 n = 3/10 M/F). Graphs show average ± SEM with statistics run using two-way ANOVA with Sidak’s multiple comparisons test. No statistical difference observed after age and gender data stratification unless signified by *p < 0.05, **p < 0.01, or ***p < 0.001. (TIF 194 kb) [file 13024_2018_293_MOESM6_ESM.tif]

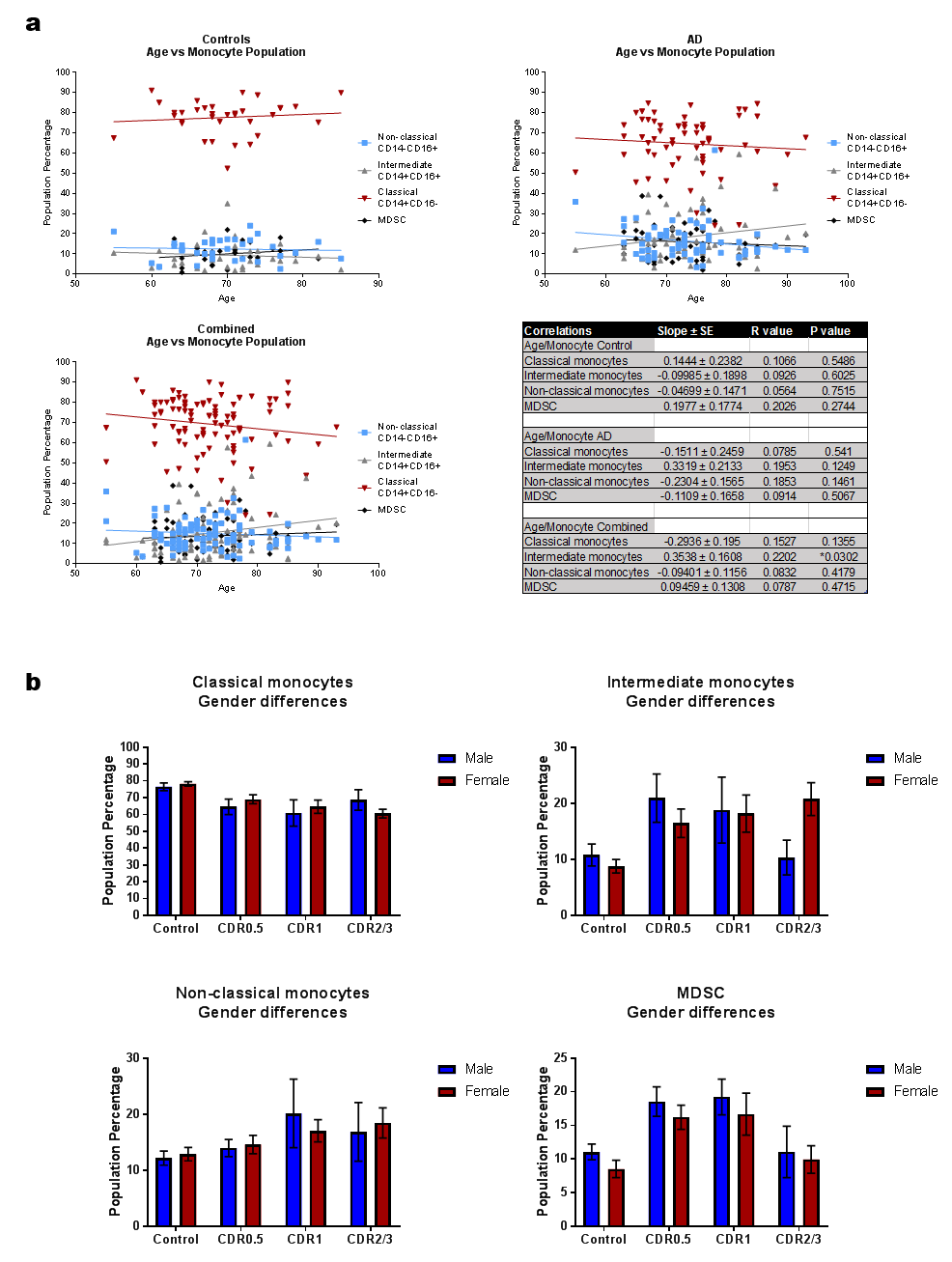

Supplement: Supplementary file 7 — Figure S5. (a) Correlation data between age and monocyte population changes. Analyses performed examined the ages of controls, varying levels of AD, and combined groups for correlations in changes in classical monocytes, intermediate monocytes, non-classical monocytes, and MDSCs (Control n = 35, AD n = 66). (b) Analyses of gender contributions to monocyte population changes among controls, CDR0.5, CDR1, and CDR2/3 (Control n = 20/15 M/F, CDR0.5 n = 15/16 M/F, CDR1 n = 8/10 M/F, CDR2/3 n = 5/12 M/F). Graphs show average ± SEM with statistics run using two-way ANOVA with Sidak’s multiple comparisons test. No statistical difference observed after age and gender data stratification unless signified by *p < 0.05, **p < 0.01, or ***p < 0.001. (TIF 246 kb) [file 13024_2018_293_MOESM7_ESM.tif]

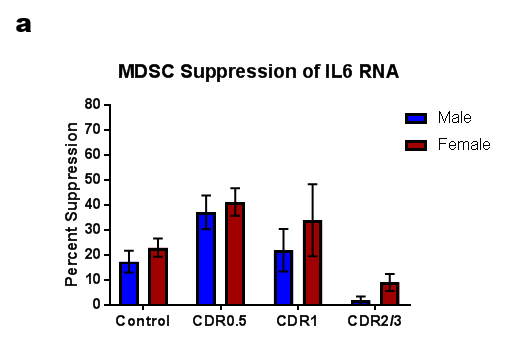

Supplement: Supplementary file 8 — Figure S7. (a) Analysis of gender contribution to MDSC suppressive function on pro-inflammatory M1 cells (Control n = 6/4 M/F, CDR0.5 n = 5/6 M/F, CDR1 n = 4/6 M/F, CDR2/3 n = 3/7 M/F). Graph shows average ± SEM with statistics run using two-way ANOVA with Sidak’s multiple comparisons test. No statistical difference observed after gender data stratification unless signified by *p < 0.05, **p < 0.01, or ***p < 0.001. (TIF 25 kb) [file 13024_2018_293_MOESM8_ESM.tif]

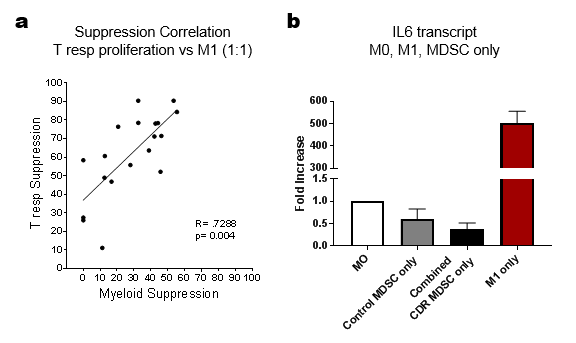

Supplement: Supplementary file 9 — Figure S8. (a) Correlation plot graphing T resp. proliferation suppression and myeloid IL-6 transcript suppression at 1:1 ratio of responding cells to MDSCs (R = .7288 p = 0.004). (b) IL-6 control experiment whereby MDSCs from controls (n = 6) and AD patients from various stages (n = 12) do not express IL-6 transcript when cultured alone in LPS/IFNγ treatments. Corroboration with no IL-6 protein in the MDSC only treated media when analyzed via ELISA (data not shown). (TIF 29 kb) [file 13024_2018_293_MOESM9_ESM.tif]
